# Overuse injuries in Swedish elite athletics– a study protocol for a prospective multifactorial cohort study

**DOI:** 10.1186/s12891-018-2296-z

**Published:** 2018-10-13

**Authors:** Andreas Lundberg Zachrisson, Pia Desai, Jon Karlsson, Elias Johanesson, Stefan Grau

**Affiliations:** 1 Center for Health and Performance, Department of Food and Nutrition, and Sport Science, University of Gothenburg, Box 300, 405 30 Gothenburg, Sweden; 2 Department of Orthopaedics at Institute of Clinical Sciences, Sahlgrenska Academy, University of Gothenburg, Göteborgsvägen 31, 431 80 Mölndal, Sweden

**Keywords:** Overuse injuries, Athletics, Biomechanics, Injury prevention, Elite sport

## Abstract

**Background:**

Overuse injuries (OI) are common in elite athletics. Previous studies have had athletes self-report injuries rather than having a medical professional provide a clear diagnosis. This might be a major reason for the inconsistencies in reported incident proportions of OI in elite athletics, in addition to the varying definitions of OI in current literature.

Risk factors or combinations of risk factors (biomechanical, clinical, and training-related) have been shown to be important in the developmental process of OI. However, no studies have examined these relationships using a multifactorial and prospective approach in elite athletics.

The purpose of this study protocol is to describe OI incidence proportion, injury severity, location, and occurrence during a complete athletics season. Moreover, possible discipline specific and injury specific risk factors that might be associated with OI will be examined.

**Methods:**

This study will be an explorative prospective cohort study including approximately 120 elite athletes. All athletes will be screened twice during one complete athletics season. The screening will consist of a body composition scan to measure muscle mass, fat free mass, lean mass, bone density, and bone mineral content. In addition, clinical examination will measure range of motion for the lower back, hip, knee, shoulder, and elbow and ankle joints. A running analysis will measure the 3D motions of the hip, knee, and ankle joints. Finally, maximal isometric strength tests of the main core and lower extremity muscles will be carried out.

To record injuries, each athlete will consult a physiotherapist or sports medicine doctor affiliated with the study to get a clear diagnosis. Injury data will be recorded according to the previously published athletics consensus statement.

**Discussion:**

Results from this study protocol will contribute more insight and detailed knowledge regarding the extent of OI occurrence among elite athletes during a complete athletics season. It will also provide further insights into which risk factors are associated with the development of OI in elite athletics.

**Electronic supplementary material:**

The online version of this article (10.1186/s12891-018-2296-z) contains supplementary material, which is available to authorized users.

## Background

Athletics is a collection of sports characterized by a high training frequency, a variable shown to be closely associated with the onset of injury [1]. Injuries are common among both amateur and elite athletes, with only minor differences in incidence rates and injury patterns [2]. The number of prevalent cases among athletes has been reported to range between 3.1 to 169.8 per 100 athletes per year [3]. Furthermore, injury incidence proportions between 50 and 76% during a complete season have been reported [1, 4, 5]. Injury incidence proportion of 68% during 1 year have been reported for Swedish elite athletics [1].

Athletes sustain most injuries during training (60% up to 91%) [5, 6], while the remaining injuries occur during competitions (9% and 40%) [3]. Lower extremities are the most commonly affected body region with reports ranging from 60 to 100% [1, 3, 6], regardless of performance level. Upper body injuries, particularly in the shoulder and arm, are most common in throwing and jumping events [1, 7, 8]. Injury patterns appear to vary between event groups; athletes in running events predominantly sustain foot, shank, and knee injuries, whereas athletes in jumping and throwing events mostly sustain knee, thigh, and lumbar injuries [1, 7, 9].

The most common type of injuries in athletics are overuse injuries (OI) (non-traumatic) [10, 11], with an incidence proportion of 96% [1]. Since overuse injuries are difficult to diagnose [12–14], a consensus statement is suggesting recording overuse injuries according to onset incident; sudden onset incident or gradual onset incident [15]. A tendon rupture is an example of a sudden onset injury, whereas Achilles tendinitis represents a gradual onset incident. Other injuries are often classified as acute (traumatic) and occur due to falls or external impact, such as hitting an obstacle (e.g. hurdles) [1].

Most injuries in athletics are classified as *severe* and lead to a break from training and competition for at least 3 weeks [1]. Previous research has shown that loss of training is a key factor for low performance, as the likelihood of achieving a performance goal increased sevenfold in athletes who completed more than 80% of planned training weeks [16]. Due to different injury surveillance techniques and definitions, a clear definition of recovery is critical to be able to record injury severity accurately [17]. The recently published athletics consensus statement for recording injuries used a time-loss definition divided into four categories spanning from 1 to 7 days to > 6 months [15].

The development of OI has been researched from different perspectives. In the past, a major area of interest has been the relationship between training load and the development of OI. Further areas of interest in this regard comprise biomechanical, clinical, and anthropometrical influences. Numerous articles have examined these variables with regard to OI development in running and other sports [1, 4, 5, 12, 13, 18].

Earlier research analyzed possible relationships between training errors and OI. Typically training volume (e.g. weekly training sessions, weekly training hours), intensity/running pace (e.g. light, moderate high or min/km), and type of training (e.g. weight training, technique training, and sprint training) were examined [1, 4, 19]. A combination of intensity and training volume (training log rank index) was shown to be associated with injury risk in elite athletics (*p* = 0.019) [1]. Overall, there is very little evidence regarding the relationship between specific training errors and the development of OI in recreational and elite athletics. One main reason for this deficit is that single training variables and their relationship to OI were investigated, neglecting possible modifying or confounding effects. More recent studies have suggested using structure-specific load capacity as a running related injury-outcome variable as it comprises more accurate quantification of running exposure [20]. A systematic review looked at information about risk factors and sex differences identifying personal factors (e.g. sex, age, BMI) and running/training related factors (e.g. training experience, number of training sessions, surface, distance and shoe use) that are associated to the onset of injury [21].

To analyze possible relationships between biomechanical variables and overuse injuries, movement analyses (sometimes in combination with the analysis of ground reaction forces) and strength measurements (isokinetic, isometric) are usually conducted, mostly for the lower extremities and the trunk. There are several retrospective studies with a focus on recreational and elite runners that have examined discrete (scalar) variables to find associations to specific OI. Suggestions of biomechanical associations from lower extremity movement/loading and strength analyses have been proposed to be associated with OI; however there are numerous conflicting results which oppose these suggestions. Several systematic reviews [22–25] have reported this dilemma and discussed that possible reasons for the conflicting results are manifold. In particular, they discuss the cause-and-effect problem. Namely, differences between healthy and injured subjects, found in studies using a retrospective design, can neither be specified as causes of, nor as a compensatory effect of an injury. Prospective study designs are considered essential to clarify cause-effect relationships and to determine interrelationships between different risk factors leading to injury [26, 27]. Further reasons for the evidence dilemma include varying kinematic models to evaluate movement, the lack of a control group, small study populations that lead to statistical underpowering, varying populations of athletes (recreational or elite), inconsistent or absent definitions of injury, and different measurement methods and study designs.

To determine possible relationships between clinical/anthropometrical variables and OI, muscle flexibility tests, range of motion (ROM) tests at the lower extremity joints, and specific clinical tests (e.g. patella compression and tilt, laxity tests of ligaments) are usually conducted. In addition, previous history of OI and anthropometric data (e.g. age, height, weight, BMI and gender) are recorded. When looking at studies exploring training variables and biomechanical risk factors, there are only a limited number of studies that have tried to find associations with overuse injuries in this category [4, 18, 28]. Previous studies have shown that gender and previous history of overuse injury are associated with the onset of new OI [1]. To date, no study to our knowledge has examined muscle flexibility and ROM and their relationship to overuse injuries in elite athletics.

The documentation of injuries in previous studies (retrospective and prospective) was based on self-reporting (e.g. questionnaires) by the athletes [1, 3–5, 14, 29, 30]. This might be a major reason for the varying incidence proportions of injuries reported in elite athletics in addition to the inconsistent injury definition used [3, 31, 32]. To ensure that future injury incidence proportions are reported accurately, injuries should be diagnosed by a medical professional, and the injury definitions developed in the consensus statement [15] should be used to accurately define injuries in elite athletics.

Methodological limitations in objective measuring of training variables are prone to be bias, as detailed and event-specific training diaries have not been used [1, 4, 5]. Moreover, documenting training over an entire season is important, as the injuries might occur in different seasonal periods (conditioning training, training camps, and competition phase) during the year.

When considering research regarding variables associated with OI in athletics (training, biomechanical, and clinical/anthropometric research), it is apparent that there are only few studies done on elite level athletes [1, 16] and no studies where multifactorial (biomechanical, clinical and training) variables were investigated at the same time. Previous research has examined associations with OI solely within one parameter (biomechanics, clinical, or training). In the past, variables expected to be associated with OI were independently tested to determine the occurrence of injury.. Variables with a statistically significant association to OI were then added to a regression model [33, 34] where “each included variable is a confounder for the outcome and is directly associated with it” [35]. However, it is questionable whether non-training-related variables (e.g. biomechanical and anthropometric variables) in themselves can lead to overuse injury [36]. The necessary cause is training thus “when studying causal mechanisms, training related characteristics should be considered as primary exposures of interest in injury research” [35]. Recent studies have introduced training load as central and most important part of the causal path to injury [20, 37]. Nevertheless, understanding mechanisms is crucial for development and successful implementation of prevention strategies for elite athletes. For example, other variables associated with OI are not taken into consideration by only modifying the training program.

The aim of the current study is to identify incidence proportion, injury severity, injury location and the occurrence of overuse injuries in elite Swedish athletics. Furthermore, possible discipline specific (e.g. distance running vs. jump events) and injury specific (e.g. foot injuries vs. hip injuries) risk factors that might be associated with injury will be examined. The purpose of this study is to add to the injury etiology in athletics and to identify athletes that are at an increased or decreased risk of injury.

### Research questions

- What are the incidence proportion, injury severity, and injury location of overuse injuries in elite Swedish athletics over a complete athletics season?
- How do personal factors (e.g. sex, age, BMI), running/training related factors (e.g. training experience, number of training sessions, surface, distance and shoe use), biomechanical factors (e.g. movement patterns, strength) and clinical factors (e.g. flexibility) relate to the occurrence of injury?
- When do overuse injuries in elite Swedish athletics occur during a season?

### Hypotheses

**H1** There will be a high incidence proportion of OI (larger than 50%) in Swedish elite athletics. Further, most OI will be of severe nature (> 28 days of; mean time-to-recovery) and will be located at the lower extremities.

**H2** Personal factors (e.g. sex, age, BMI), running/training related factors (e.g. training experience, number of training sessions, surface, distance and shoe use), biomechanical factors (e.g. movement patterns, strength) as well as clinical factors (e.g. flexibility) will be related to the occurrence of injury.

**H3** OI in elite Swedish athletes will occur more frequently in the beginning of the season and during training phases with high training volume (e.g. follow up kilometers, follow up training hours/training sessions) compared to seasonal periods with low training volume controlling for personal, running/training related, biomechanical and clinical factors.

## Methods/design

### Design

This project is designed as an explorative prospective cohort study over a 1 year period. All athletes will be screened twice (spring/autumn) to collect biomechanical and clinical data. The screening protocol for each athlete will depend on the event in which they compete (Table 1). Furthermore, daily training data will be collected for each athlete. All scientific articles from this research project will follow the STROBE statement [38].

**Table 1:** Overview of screening tests for each event group

	Triple jump/Long jump	High jump	Pole vault	Sprint	Middle/Long distance	Javelin	Discus	Shot put	Hammer throw
iDXA	X	X	X	X	X	X	X	X	X
Clinical examination	X	X	X	X	X	X	X	X	X
Running analysis	X			X	X				
Knee stability	X	X	X	X	X	X	X	X	X
Strength	X	X	X	X	X	X	X	X	X

### Participants

The main prerequisite for participation and to fulfil our definition of an elite athlete, participants must have placed in the top six of the senior national championships or top three of the youth national championships between 2015 and 2017. Athletes must be a member of a registered athletics club in Gothenburg, Sweden, and be healthy and able to perform all tests with no restrictions at the initial screening point as confirmed by the project’s physiotherapist. A list of eligible male and female elite athletics athletes will be compiled by Gothenburg Athletics Federation (GFIF) and sent by email with an invitation for participation in the project. Approx. 120 athletes will be invited to participate in the study, and an information meeting will be held for both athletes and coaches regarding the content and setup of the project. The invited athletes will vary in disciplines, including distance running (800 m up to marathon), sprint (60 m up to 400 m, incl. hurdles), jumping (high jump, pole vault, triple jump, and long jump), and throwing (javelin, hammer throw, shot put, and discus) events.

### Injury definitions and classifications

An athletics injury will be defined as follows:

*Any musculoskeletal pain felt during athletics training or competition that inflicted a non-voluntary reduction or complete stop from athletics training for at least 24 h, and was diagnosed by a trained medical professional,* e.g. *a physiotherapist and/or sports medicine doctor.*

The categorization of different injuries will be based on a previous consensus statement regarding injury data collection in epidemiological athletics studies, where it was decided to classify injuries according to the onset incident [15].

All recorded injuries will be divided into four categories according to injury location: Foot/shank, knee, thigh, and upper body injuries. A clear injury diagnosis will be made by a medical professional. A previously injured athlete will be considered injury-free when reporting full return to full athletics training.

### Injury severity

Time-loss from athletics training and competition will be used to quantify injury severity. Injury severity will be classified into four categories: minor (1–7 days), moderately serious (8–28 days), serious (> 28 days-6 months), and long-term (> 6 months) [11, 17]. Quantification of time-loss will be stopped when the injured athlete returns to full athletics training according to the training documentation they submit monthly.

### Injury data collection

All athletes will use a mobile phone application on a daily basis where they can report whether they have felt any pain or have suffered an injury that affected their regular training. The project leader will contact any athlete reporting pain or injury to gain information on whether they have sought medical attention. Each athlete will consult a physiotherapist or sports medicine doctor affiliated with the study if injured. If they already have a support team, the injury data will be collected from the external medical professional. An additional verification will be performed using information from the athletes’ mobile phone application, direct contact with the athletes, and/or by talking to them and their respective coach. Only injury data that matches the injury definition will be recorded.

### Clinical examination

All clinical examinations, measurements of passive range of motion (ROM) for the lower back, hip, knee, shoulder, and elbow and ankle joints will be performed by an experienced physiotherapist according to the neutral-zero-method [39] using a measurement device (Mobee Fit/Med) comprised of an accelerometer, gyroscope, and magnetic field sensor (SportMed A.G. SA, Bitburg, Germany). The examination will be performed with the athlete lying in a supine or prone position, or lying on their side. The following measurements will be performed for all athletes: hip flexion, hip extension, hip abduction, hip adduction, hip internal rotation, hip external rotation, knee flexion, and knee extension. All athletes will perform ankle dorsiflexion and ankle plantarflexion except for throwers. The following additional tests will be performed for throwers: shoulder flexion, shoulder extension, shoulder external rotation, shoulder internal rotation, rotation of the thoracic/lumbar spine, elbow flexion, and elbow extension. The maximum angular value from three repetitions will be recorded for all joint movements.

### Running analysis

All athletes (except throwers and high jumpers/pole vaulters) will run with standardized neutral running shoes on a treadmill at a given controlled speed: 18 km/h (middle/long distance runners) or 21 km/h (sprint and long jump/triple jump). Familiarization with the lab environment, surface, and running speed will take place prior to testing, enabling the athletes to recreate their natural running style. All measurements will be recorded with a 3D motion capture system (Qualisys AB, Gothenburg, Sweden), consisting of 16 cameras with infrared light at a sampling frequency of 400 Hz [40]. Participants will be equipped with 34 retroreflective spherical markers attached on specific anatomical landmarks according to international guidelines [41, 42].

The following movement variables will be evaluated during stance: hip adduction range of motion, hip adduction velocity, knee flexion range of motion, knee flexion velocity, rear foot pronation range of motion, rear foot pronation velocity, ankle plantar−/dorsiflexion range of motion, and sagittal touch down angle of the foot towards the ground. Motions of the hip, knee, and ankle joints will be calculated relative to the neutral standing position. The mean values will be based on 10 consecutive strides.

### Isometric strength tests

Isometric maximum strength tests will be performed to measure strength for trunk and lower extremity muscles, and will be performed on isometric testing devices (David Health Solutions Ltd., Helsinki, Finland). All measurements will be performed according to a standardized test protocol (Additional file 1). The following maximal isometric strength measurements will be tested: trunk extension, trunk flexion, trunk rotation, hip abduction, hip adduction, knee extension, and knee flexion.

Calculations for the following strength balance ratios will be performed: trunk flexion:extension, trunk rotation right:left, hip abduction:adduction, knee extension left:right, knee flexion left:right, and knee flexion:extension [40].

All subjects will have time to familiarize themselves with the devices. Warmup will consist of the subjects first performing dynamic exercises against an increasing resistance followed by isometric sub-maximal contractions. Trunk flexion will be tested at 0°, trunk extension at 30°, and trunk rotation at 30° on the left and right sides. Hip abduction and adduction will be tested bilaterally in a hip abduction angle of 15° in each hip. Knee flexion and extension will be tested unilaterally at 30° and 60° for knee flexion and extension, respectively. Subjects will be seated and secured with a safety belt, and no self-stabilization will be permitted during the measurements. Two maximal isometric contractions will be performed with a minimum of 30 s of rest between them. If the difference between the first two tests exceeds 10%, a third test will be conducted and the maximal torque value will be documented. Verbal encouragement will be used by the test leader to increase the likelihood of the test subjects reaching their maximal strength potential.

Additionally, isometric maximum strength tests will be performed for hip extension and hip flexion on the IsoMed 2000 (D&R GmbH, Hemau, Germany) according to the same standardized procedure. Instead of being in a seated position, subjects will perform the tests in a supine position. Hip extension and flexion will be tested at 40°.

All maximum isometric strength values will be normalized to body weight.

### Knee laxity

All athletes will perform a one leg squat to assess knee laxity [43]. Each athlete will perform three trials per leg. Knee adduction movement in centimeters will be measured unilaterally for both legs. The knee laxity test will be recorded by the 3D motion capture system.

### Body composition scan

All athletes will undergo a low dose radiological body composition scan (Lunar iDXA, GE Healthcare, USA). Muscle mass, fat free mass, lean mass, bone density, and bone mineral content will be measured and evaluated by experienced technicians. The measurement protocol will follow guidelines outlined in a previous study regarding positioning and standardization [44, 45].

### Training data collection

Training diaries were created together with the coaches for four athletic categories: middle/long distance, sprint, throw, and jump. All training diaries were divided into different thematic columns categorizing the type of training conducted (Figs. 2, 3, 4, 5, 6, 7 and 8). Each athlete will fill out the training diary on a daily basis, and subsequently send it to the project leader at the end of each month.

### Statistical analysis

Analyses will be performed for all eligible athletes that are injury free at baseline. All injuries will be represented, e.g. one athlete can have multiple injuries during the season and be represented in more than one injury location category. Injury severity and injury location data will be presented using descriptive statistics. Categorical data will be presented in terms of frequency and proportion (%). Athletes will be divided according to event group and injury severity or injury location.

The relationship between OI and personal, running/training related, biomechanical and clinical factors will be assessed by correlational analysis. Depending on the scale level of the variables, tetrachoric, polychoric and polyserial correlations will be used.

The analysis of time to OI will use cumulative training volume. The event variable will be OI (coded 1 for injury, 0 for non-injury). Once a subject had presented and was assessed by a medical doctor with an injury his/her survival time will be considered to be terminated. Hence, any bias related to further presentation with the same injury, or with an injury secondary to the first, is eliminated. Examples of other reasons for censoring will be: not reporting training data, disease, pregnancy or other nonrelated OI causes that lead to a permanent cessation of training. The proportion of OI as a function of training volume will be calculated using the product-limit method (i.e., Kaplan-Meier).

**Fig. 1:**
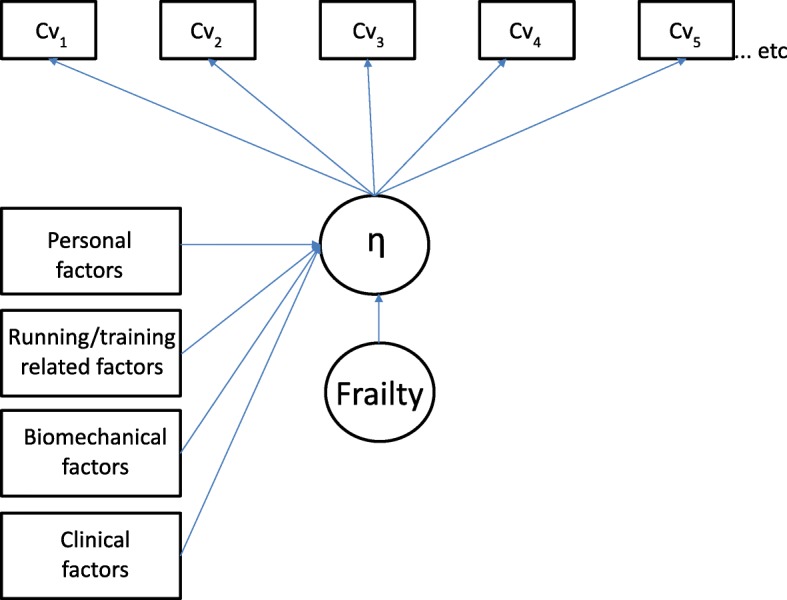
Cumulative training volume related injury path diagram. Survival model with time-invariant covariates and single latent class variable

In survival analysis it is often important to take unobserved heterogeneity into account among the subjects. This implies that unobserved covariates or random error can bias the main effect parameters and the standard errors if those are taken explicitly into account [46]. In continuous-time survival analysis unobserved heterogeneity is modelled by the use of frailties. The frailty parameter represents the heterogeneity by random effects (i.e., continuous latent variables). Two models will be estimated; one without the frailty parameter and one with the frailty parameter in order to assess if unobserved heterogeneity has an impact on the main effect parameter (Fig. 1). OI as a function of cumulative training volume is captured by the latent variable, η. The relationship between this latent variable and personal, running/training related, biomechanical and clinical factors will be examined.

A probability of less than 5% was considered significant for a priori hypotheses. H1 will be evaluated using SPSS statistics (Version 25, IBM Inc., Armonk, New York) and H2 and H3 will be evaluated using Mplus version 8 (Muthén and Muthén, 1998–2017). Significance will be accepted at *p* < 0.05.

## Discussion

This study protocol will partly act as a verification of previously published studies within the field of athletics, where high incidence proportions were reported. Additionally, it is the first study in the field of athletics that does not rely on self-reporting of injury by the athletes, but instead on a clear diagnosis for each injury, made by a medical professional. Should the incidence proportion prove to be similar or higher than previously reported, the problematic nature of injuries suffered by elite athletes in athletics will be highlighted.

Moreover, this protocol will be a first step toward implementing/utilizing a multifactorial approach to define potential risk factors in the development of overuse injuries in Swedish elite athletics. As stated in a previous study [47], screening protocols are difficult to implement due to the multifactorial nature of athletics. Nevertheless, no previous study has included biomechanical, clinical, anthropometrical, and training data collaboratively to examine associations with injury-outcome. The combination of these injury-outcome associations could give new insight into the development of overuse injuries in elite athletics. The proposed study protocol will be the first study that follows the published athletics consensus statement [15] regarding the classification of injuries according to onset incidence.

To examine injury occurrence during the course of a complete athletics season, several aspects must be taken into consideration. As there are slight differences in season layout/planning between groups, adjustments must be made to ensure that all event groups are covered in the same seasonal periods. Furthermore, injury occurrence will be possible to track and document. It will also be determined whether the injuries tend to occur during the conditional periods, when the athletes normally have a higher training volume, or during competition periods, when maximal performance is required. In combination with potential risk factors and training information, injury occurrence could help coaches and athletes plan and adjust their training and competition schedule to try and minimize the risk of injury.

To increase the sample size and to mitigate effects from dropouts the study protocol could be continued for more than 1 year, continuously adding athletes to the study. With enough athletes, specific intervention programs could be implemented for specific categories of athletes (e.g. high injury risk athletes) to determine whether specific risk factors can be avoided or decreased.

Another important aspect is the possibility that previously described single risk factors (e.g. amount of pronation, amount of training) might not be predictive of general or specific injury onset in every athlete or discipline, as it might be the specific combination of risk factors that could give an indication of injury risk. To account for this, the implemented screening protocol includes all previously identified single factors (biomechanical, clinical, anthropometric, and training load) that seem to be risk factors for overuse injuries.

**Fig. 2:**
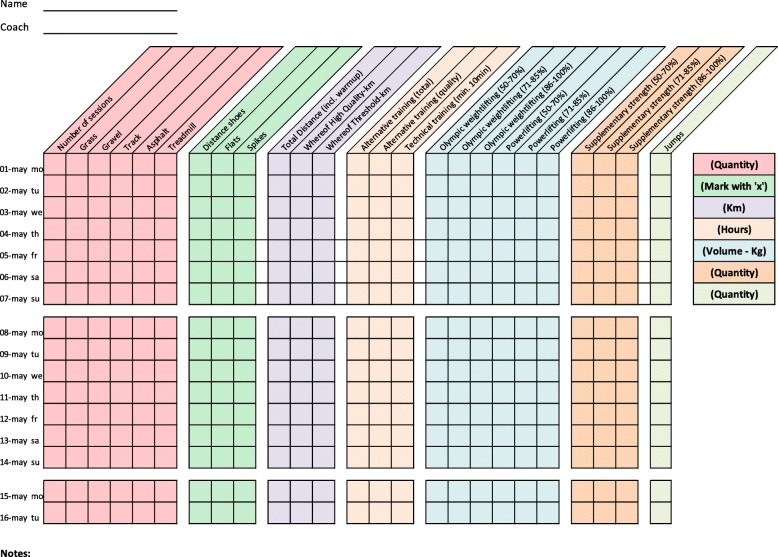
Example of training diary for middle/long distance runners

**Fig. 3:**
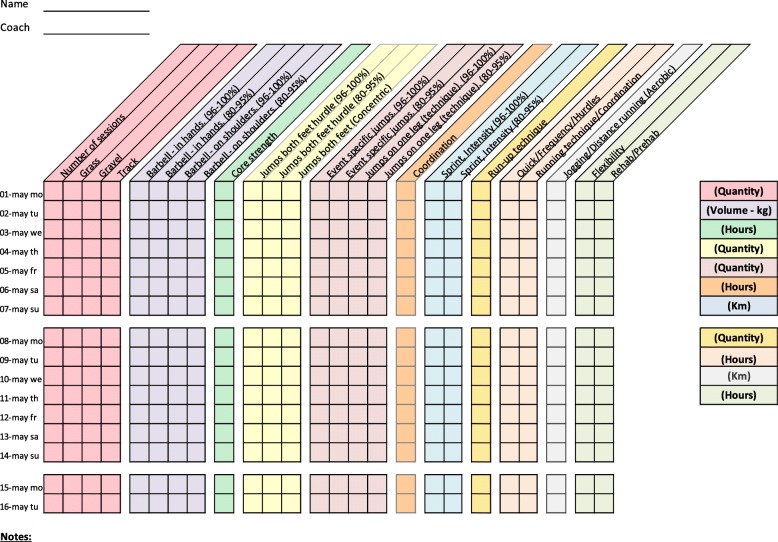
Example of training diary for jumping events

**Fig. 4:**
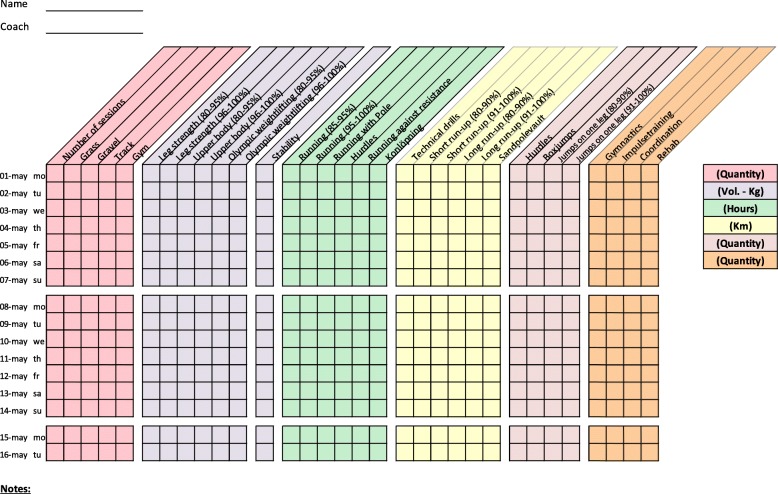
Example of training diary for pole vault

**Fig. 5:**
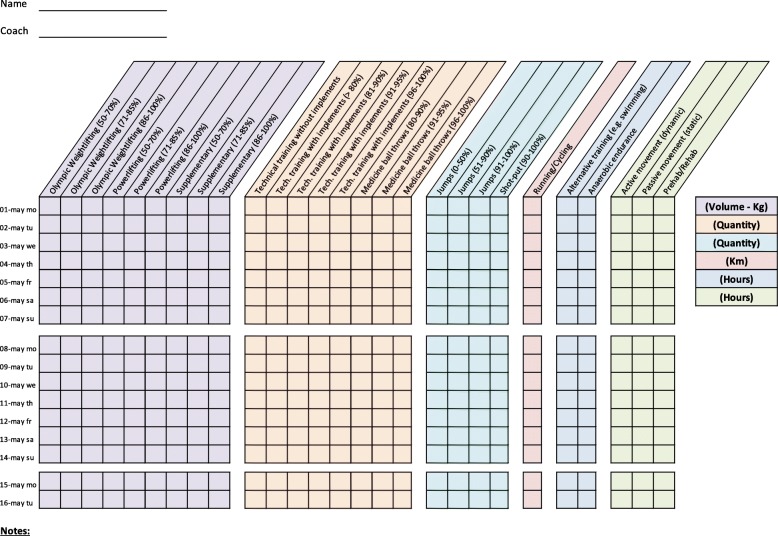
Example of training diary for throwing events

**Fig. 6:**
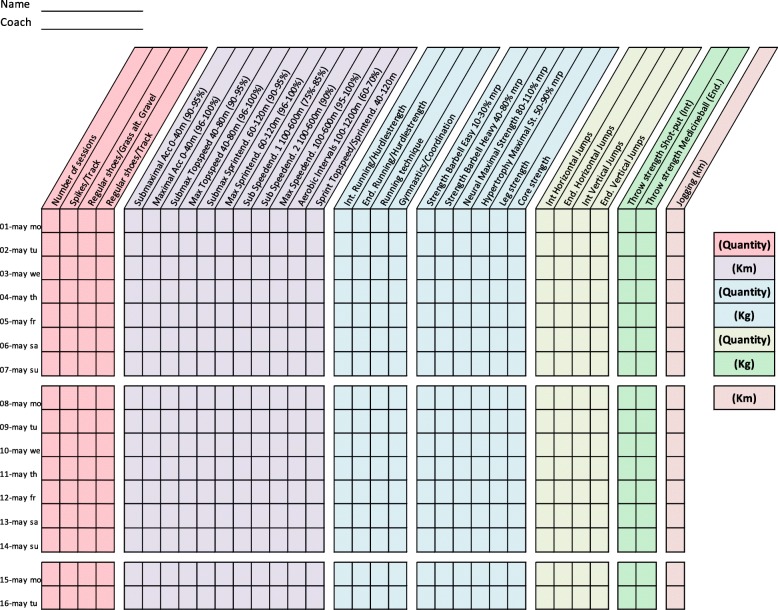
Example of training diary for long sprint

**Fig. 7:**
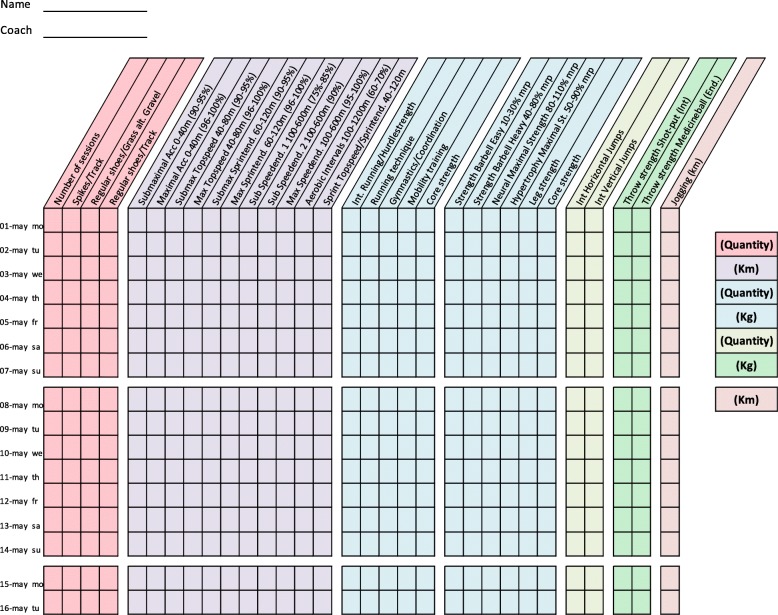
Example of training diary for short sprint (hurdles)

**Fig. 8:**
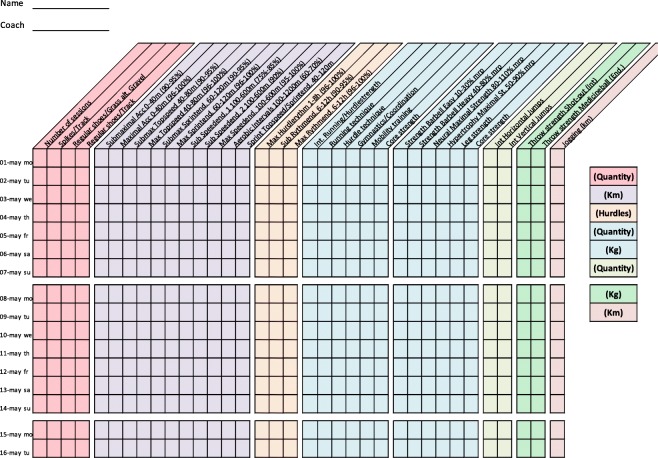
Example of training diary for short sprint

Furthermore, it is important that all athletes submit accurately completed training diaries (Figs. 2, 3, 4, 5, 6, 7 and 8), and that all athletes and coaches have agreed on how to fill in each category (e.g. strength or technique training) in the same manner. Failure to do so may result in biased comparisons between athletes and event groups, and lead to incomplete or inaccurate results.

## Additional file

Additional file 1: Standardized test protocol for isometric strength tests. (DOCX 55 kb)

## Abbreviations

BMI: Body mass index
GFIF: Gothenburg Athletics Federation
OI: Overuse injury
ROM: Range of motion
