# Supplementary material for: Overuse injuries in Swedish elite athletics– a study protocol for a prospective multifactorial cohort study
Source: BMC Musculoskelet Disord. 2018 Oct 13;19:370. doi: 10.1186/s12891-018-2296-z (PMC6186111; doi:10.1186/s12891-018-2296-z)
Supplement: Supplementary file 1 — Standardized test protocol for isometric strength tests. (DOCX 55 kb) [file 12891_2018_2296_MOESM1_ESM.docx]

CASE REPORT FORM - Testcenter

**MUSCULAR STRENGTH & RANGE OF MOTION - DAVID**

**
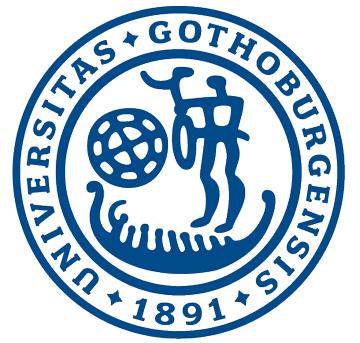
**

| Name | | | |  |  |  |  |  |  |  |  |  |  |  |  |
| --- | --- | --- | --- | --- | --- | --- | --- | --- | --- | --- | --- | --- | --- | --- | --- |
|  | | | |  |  |  |  |  |  |  |  |  |  |  |  |
|  | | | | |  |  |  |  |  |  |  |  |  |  |  |
| Height: | | | |  |  | m | Weight: | | |  | | kg |  |  |  |
|  | | | |  |  |  |  |  |  |  |  |  |  |  |  |
|  | | | | |  |  |  |  |  |  |  |  |  |  |  |
| Birthdate | | | |  |  |  |  |  |  |  |  |  |  |  |  |
|  |  | | |  |  |  |  |  | **STRENGTH** | | |  | **ACTIVE ROM** | |  |
|  | **KNEE** | |  | |  |  |  |  |  |  |  |  |  |  |  |
|  | | | | |  |  |  |  |  |  |  |  |  |  |  |
| David 200 | | | | | **Extension** | | right |  |  |  |  | N | - | ° | Seat: …………… |
|  |  |  |  |  | at 60° | |  |  |  |  |  |  |  |  |  |
|  |  |  |  |  |  |  |  |  |  |  |  |  |  |  |  |
|  |  |  |  |  |  |  |  | |  |  |  |  |  |  |  |
|  |  |  |  |  |  |  | left |  |  |  |  | N | - | ° | Foot: …………… |
|  | | | | |  |  |  |  |  |  |  |  |  |  |  |
|  | | | | |  |  |  | |  |  |  |  |  |  |  |
| David 300 | | | | | **Flexion** | | right |  |  |  |  | N |  | ° | Seat: …………… |
|  |  |  |  |  | at 30° | |  |  |  |  |  |  |  |  |  |
|  |  |  |  |  |  |  |  |  |  |  |  |  |  |  |  |
|  |  |  |  |  |  |  | left |  |  |  |  | N |  | ° | Foot: …………… |
|  | **HIP** |  | | |  |  |  |  |  |  |  |  |  |  |  |
|  |  |  |  |  |  |  |  |  |  |  |  |  |  |  |  |
|  | | | | |  |  |  |  |  |  |  |  |  |  |  |
| David 310 | | | | | **Abduction** | |  |  |  |  |  | N |  | ° |  |
|  |  |  |  |  |  |  |  |  |  |  |  |  |  | |  |
|  |  |  |  |  | at 15° | |  |  |  |  |  |  | ouside / abd | |  |
| David 320 | | | | | **Adduction** | |  |  |  |  |  | N |  |  |  |
|  | **UPPER BODY** | | | |  |  |  |  |  |  |  |  |  |  |  |
|  |  |  |  |  |  |  |  |  |  |  |  |  |  |  |  |
|  | | | | |  |  | | |  |  |  |  |  |  |  |
| David 110 | | | | | **Back Extension** | | |  |  |  |  | N | - | ° | Seat: …………… |
|  |  |  |  |  | at 30° | |  |  |  |  |  |  |  |  |  |
|  |  |  |  |  |  |  |  |  |  |  |  |  |  |  |  |
|  | | | | |  |  | | |  |  |  |  |  |  |  |
| David 130 | | | | | **Abd.Flexion** | | |  |  |  |  | N |  | ° | Foot: …………… |
|  |  |  |  |  | at 0° | |  |  |  |  |  |  |  |  |  |
|  |  |  |  |  |  |  |  |  |  |  |  |  |  |  |  |
|  | | | | |  |  |  | |  |  |  |  |  |  |  |
| David 120 | | | | | **Rotation** | | right |  |  |  |  | N |  | ° | Seat: …………… |
|  |  |  |  |  |  |  | |  |  |  |  |  |  |  |  |
|  |  |  |  |  | at -30° / 30° | | | | | | |  |  |  |  |
|  |  |  |  |  |  |  |  | |  |  |  |  |  |  |  |
|  |  |  |  |  |  |  | left |  | | | | N | - | ° | Foot: …………… |
|  |  |  |  |  |  |  |  |  |  |  |  |  |  |  |  |
